# Spatial Navigation and the Central Complex: Sensory Acquisition, Orientation, and Motor Control

**DOI:** 10.3389/fnbeh.2017.00004

**Published:** 2017-01-24

**Authors:** Adrienn G. Varga, Nicholas D. Kathman, Joshua P. Martin, Peiyuan Guo, Roy E. Ritzmann

**Affiliations:** ^1^Department of Biology, Case Western Reserve UniversityCleveland, OH, USA; ^2^Department of Biology, Colby CollegeWaterville, ME, USA

**Keywords:** central complex, spatial navigation, head direction cells, population code, motor activity, reflex

## Abstract

Cockroaches are scavengers that forage through dark, maze-like environments. Like other foraging animals, for instance rats, they must continually asses their situation to keep track of targets and negotiate barriers. While navigating a complex environment, all animals need to integrate sensory information in order to produce appropriate motor commands. The integrated sensory cues can be used to provide the animal with an environmental and contextual reference frame for the behavior. To successfully reach a goal location, navigational cues continuously derived from sensory inputs have to be utilized in the spatial guidance of motor commands. The sensory processes, contextual and spatial mechanisms, and motor outputs contributing to navigation have been heavily studied in rats. In contrast, many insect studies focused on the sensory and/or motor components of navigation, and our knowledge of the abstract representation of environmental context and spatial information in the insect brain is relatively limited. Recent reports from several laboratories have explored the role of the central complex (CX), a sensorimotor region of the insect brain, in navigational processes by recording the activity of CX neurons in freely-moving insects and in more constrained, experimenter-controlled situations. The results of these studies indicate that the CX participates in processing the temporal and spatial components of sensory cues, and utilizes these cues in creating an internal representation of orientation and context, while also directing motor control. Although these studies led to a better understanding of the CX's role in insect navigation, there are still major voids in the literature regarding the underlying mechanisms and brain regions involved in spatial navigation. The main goal of this review is to place the above listed findings in the wider context of animal navigation by providing an overview of the neural mechanisms of navigation in rats and summarizing and comparing our current knowledge on the CX's role in insect navigation to these processes. By doing so, we aimed to highlight some of the missing puzzle pieces in insect navigation and provide a different perspective for future directions.

## Introduction

Insects are by just about any measure the most successful animal group inhabiting almost every conceivable niche on the planet. Behavioral repertoires range from slow walking (e.g., stick insects) to rapid flying (e.g., houseflies). Some species undergo remarkable migrations across entire continents (e.g., monarch butterflies) while others move purposefully within smaller ranges (e.g., dung beetles). Insects are effective predators (e.g., dragonflies and praying mantis), harvesters (e.g., honeybees) and foragers (e.g., cockroaches). Each of these animals must deal with changing environmental and internal conditions. Some dung beetles move only at night while other species are diurnal (el Jundi et al., 2015). Predators may change their stalking behaviors as they become satiated (Holling, 1966; Inoue and Matsura, 1983). The recent explosion of data on the central complex (CX) (Pfeiffer and Homberg, 2014) has documented numerous types of sensory information that converge in these midline neuropils. Moreover, large amounts of neuromodulatory receptors and targets have been identified (Kahsai and Winther, 2011; Boyan and Liu, 2016) and motor control effects demonstrated (Bender et al., 2010; Martin et al., 2015). These studies combine to suggest that the CX plays a pivotal role in guiding appropriate behaviors for each species and adjusting the accompanying movements to match the current context that an individual insect finds itself in at any point in time. In this review, we will primarily focus upon our laboratory's work on the role of CX circuits in controlling navigation in one successful insect, the cockroach *Blaberus discoidalis*, while also discussing relevant findings in other insects. One reason for focusing on cockroaches is that they occupy an ecological niche similar to rat habitats, which are a major model for mammalian navigation. Both rats and cockroaches are scavengers that forage in darkened environments and often navigate in complex, maze-like burrows (Roth and Willis, 1960; Feng and Himsworth, 2014). As they move, they must seek out targets such as food items or potential mates while navigating through complex terrains and avoiding predators (Meyer et al., 1981; Okada and Toh, 1998; Canonge et al., 2009). Whether these shared ecological and behavioral traits similarly influenced the neural mechanisms governing navigation in these distant species, is not known.

In a previous review (Ritzmann et al., 2012), we described movements that the cockroach makes in a large, well-lit arena as they seek out darkened shelters. Because cockroaches have a strong tendency to remain near walls, but greatly prefer the dark, we expected individuals to wall-follow until they detected the dark shelter then move directly toward that part of the arena (Daltorio et al., 2013). The paths that they took did not support that hypothesis. Instead, they appeared to move randomly through the arena but, indeed, did end up in the shelter. A closer analysis of the paths indicated that the cockroaches did take less time to reach the shelter than to reach the same area without a dark shelter present, indicating efficient goal-directed navigation. They also stayed in the darkened shelter for a longer period of time than they did in any comparable region of the arena, suggesting that the seemingly random path was in fact targeting the shelter.

An algorithm, called RAMBLER, simulated the movements of the insect quite well (Daltorio et al., 2013). Under this scheme, a simulated cockroach evaluates whether it is still in contact with the wall and whether it can still see the dark shelter. In live insect observations, cockroaches tend to increase walking speed when they move away from a wall toward the center of the arena (Bender et al., 2011), possibly to reduce the time spent in the open. If the shelter was behind the cockroach, the probability that it would turn increased and the animal tended to turn back to the place where it last detected the shelter. The RAMBLER algorithm captured these properties and implied that some sophisticated decisions might be made in higher centers. Several factors implicated the CX in that role. First, electrolytic lesions in the cockroach CX increased the number of “wrong” turns made while walking on a track (Harley and Ritzmann, 2010). Second, recordings in the CX clearly demonstrated antennal responses that encoded the direction and velocity and antennal deflections (Ritzmann et al., 2008). Finally, CX activity recorded in tethered cockroaches demonstrated increases in firing rate that preceded changes in walking speed, while stimulation through the same electrodes evoked speed changes (Bender et al., 2010). In this review, we will describe findings in our laboratory and others that not only suggest that the CX is involved in this kind of navigation but begin to outline what that role might be.

## Sensory inputs to the CX

Many different types of sensory information project to the CX and many will undoubtedly be described in detail in other papers in this special issue. Included in this list are polarized light (Heinze and Homberg, 2007; Sakura et al., 2008; Heinze et al., 2009), mechanical deflection of the antenna (Ritzmann et al., 2008) and various forms of non-polarized signals (Heinze and Reppert, 2011; Rosner and Homberg, 2013; Seelig and Jayaraman, 2013; Kathman et al., 2014; Bockhorst and Homberg, 2015). For the goal-directed navigational task outlined above, antennal and visual cues appear to be very important.

Many insects use mechanical cues from antennal contact to guide movements. In stick insects, gap crossing behavior is initiated when the antennae detect a gap in the substrate they are walking on and are further guided by searching front leg movements (Bläesing and Cruse, 2004). Leg movements associated with turning in the stick insect are also guided by antennal contact (Dürr et al., 2001; Dürr and Ebeling, 2005), through descending pathways from the brain to the thoracic ganglia (Ache et al., 2015). The descending pathways bypass higher processing areas such as the CX. Nevertheless, it is reasonable to expect that parallel branches also reach the CX. In cockroach, the antennae clearly are used in initiating climbing behaviors over substantial blocks, since ablation of part or all of the antennae affect the onset time of the climb accordingly (Harley et al., 2009). Lesions in specific regions of the CX compromise either climbing or turning behaviors indicating that the CX plays a role in utilizing mechanosensory information during navigation (Harley and Ritzmann, 2010).

**Figure 1:**
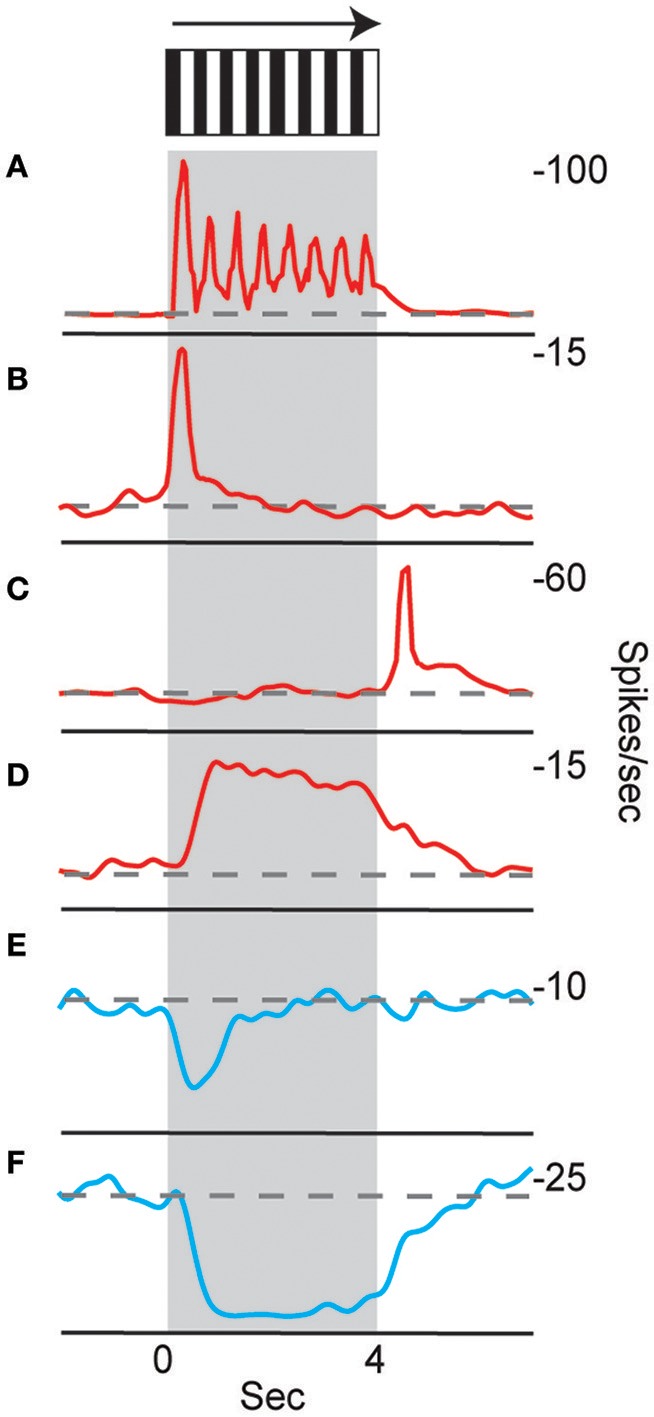
**Temporal properties of wide-field motion responses**. Six response types were found **(A–F)**. These include **(A)** units with spiking entrained to the temporal frequency of the grating, phasic excitatory responses at the beginning **(B)** and end **(C)** of movement, **(D)** tonic excitatory response lasting the duration of movement, and inhibitory phasic **(E)** and tonic **(F)** responses. Examples of all response types were found for both directions of movement, despite only responses to right movement being shown. Gray block indicates duration of stimulus and dashed line indicates baseline firing rate (Kathman et al., 2014).

**Figure 2:**
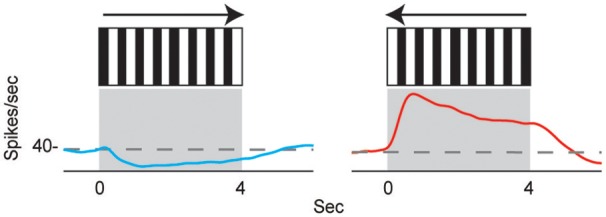
**Directional selectivity**. Units were directionally selective, often with showing directional opponency, with inhibitory responses to one direction of motion, and excitatory responses to the other (Kathman et al., 2014).

Neurons in the CX of several insects have been shown to respond to visual cues (Ritzmann et al., 2008; Heinze and Reppert, 2011; Rosner and Homberg, 2013). Visual feature detection was demonstrated in *Drosophila* using two-photon calcium imaging of neural activity in genetically-targeted CX populations (Seelig and Jayaraman, 2013). The responses of CX ring neurons resemble those in the mammalian primary visual cortex in that they are retinotopically arranged and have visual fields comprised of excitatory and inhibitory subfields. Moreover, these ring neurons were found to have strong and often direction sensitive responses. In addition to polarized light responses, monarch butterflies, dung beetles and locusts were also shown to be sensitive to non-polarized light stimuli at specific azimuthal orientations (Heinze and Reppert, 2011; el Jundi et al., 2014, 2015). In cockroach, extracellular recordings revealed CX neurons that respond to both antennal stimulation and light changes (Ritzmann et al., 2008). More recently, wide field visual stimuli were further evaluated (Kathman et al., 2014). For these latter studies, cockroaches were restrained in a tube and a 16 channel silicon probe was inserted into the CX in a variety of places in either the fan-shaped body (FB) or the ellipsoid body (EB). Vertically oriented grating patterns with variable direction, speed, and stripe width were projected onto a screen in front of the insect to simulate the yaw rotation, or turning, of the animal. These stimuli produced a wide range of CX responses including phasic and tonic excitation as well as inhibition (Figure 1). Phasic responses occurred either at the onset of stimulus presentation or at the termination of the visual stimulus. Tonic responses often were directional (Figure 2). That is, some units were excited by left moving grating patterns but either did not respond, responded at significantly lower levels or were inhibited by right moving stimulation. In the same recording both left biased and right biased neurons were found.

**Figure 3:**
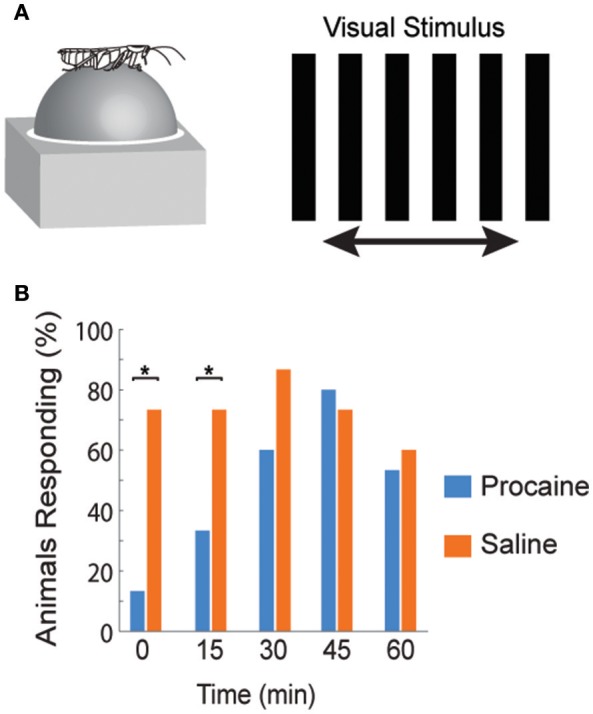
**Optomotor response is reduced after reversible chemical ablation in the CX. (A)** Turning response of *Blaberus discoidalis* to shifting stripes was measured while the insect was tethered to an air-supported Styrofoam ball. **(B)** Proportions of animals with a successful optomotor response at 15 min time intervals after the injection of procaine (blue) or saline (orange) into the CX. Procaine injected animals were significantly different (χ2 test, *P* < 0.05) from saline controls at 0 and 15 min (^*^) and recovered by 30 min (Kathman et al., 2014).

The directional responses of tonic CX neurons suggested that turning movements could be controlled at least in part by CX circuitry. Its role in optomotor responses was tested by injecting the local anesthetic, procaine, into the CX (Kathman et al., 2014). The cockroaches were tethered over an air suspended ball and grating patterns moving left or right were projected in front of the animals. As the cockroach walked on the ball its movements were monitored with optical sensors. The pattern of moving stripes readily generated optomotor responses in the direction of the stripes' movement, presumably in an attempt to stabilize the insect's visual field. Procaine is a voltage sensitive K^+^ and Na^+^ channel blocker (Devaud et al., 2007) that silences action potentials but only for short periods of time. To verify its effect in the CX, we injected procaine into the CX of restrained cockroaches while recording neural activity. Action potentials in the region where procaine was injected were completely silenced for 20 min and returned to baseline firing rates at about 30 min post-injection. Regions outside the CX were unaffected. When procaine was injected into the CX of tethered cockroaches, the optomotor responses decreased significantly then returned following the same time course as that found in silencing CX neurons (Figure 3). Similar injections with saline had no effect.

The optomotor observations suggest that CX neurons are involved in guiding movements in response to wide field visual stimuli. Additionally, several studies suggested that visually guided behaviors in the CX are context dependent. For instance, feature detection responses of some EB neurons were diminished in flight, but not during walking (Seelig and Jayaraman, 2013), while a group of FB neurons were shown to be unresponsive while the fly was quiescent but responded to translational optic flow during flight (Weir et al., 2014). Similar context dependent sensory processes could shape the cockroach's behavior as it moves in the arena and executes turns.

## What can we learn from rat navigation studies?

As the cockroach moves through its environment in ways that are similar to the arena experiment described above, information on where it currently is and how it got there play an important role. Yet the neural dynamics underlying such navigational processes in insects are not well understood. On the other hand, several decades of research on mammalian navigation circuits provide us with some basic theories to test. The majority of these studies used rats as a model animal (McNaughton et al., 2006; Jacobs and Menzel, 2014; Geva-Sagiv et al., 2015). The demands of navigation are similar for rats and cockroaches: they are nocturnal, tend to move through restricted corridors and rely heavily on both visual and tactile cues (Feng and Himsworth, 2014). Their similar ecology and foraging behaviors indicate that the two model organisms likely depend on the same sensory cues and similar integration processes to orient themselves, thus we predict that there might be some similarities between the circuits underlying navigation. In this section, we present a simplified outline of rat navigation circuits and a general description of spatial cell types to introduce some of the main concepts that are necessary for navigation—at least in mammals. Considering our limited understanding of associative processes in the insect brain, rather than examining the information flow from sensory perception to motor control, we will restrict this section to the abstract representation of spatial and contextual information in the rat brain, which we will refer back to in the section concerned with the CX's role in navigation.

Spatial navigation in all animals requires the integration of both external and internal sensory information (Geva-Sagiv et al., 2015). External sensory cues - also called allothetic cues - are visual, olfactory, auditory and tactile/mechanosensory information about the environment external to the body. Internal sensory cues—also called idiothetic cues—are derived from self-motion in the form of vestibular cues (or mechanosensory cues), optic flow, proprioceptive feedback and motor efference copy from the limbs. These sensory cues get integrated and compressed to form an inner representation of the environmental and behavioral context. This information can then be used in motor centers to induce and shape optimal motor commands that lead to successful navigation in that particular context (Moser et al., 2008; Hartley et al., 2014; Schiller et al., 2015).

**Figure 4:**
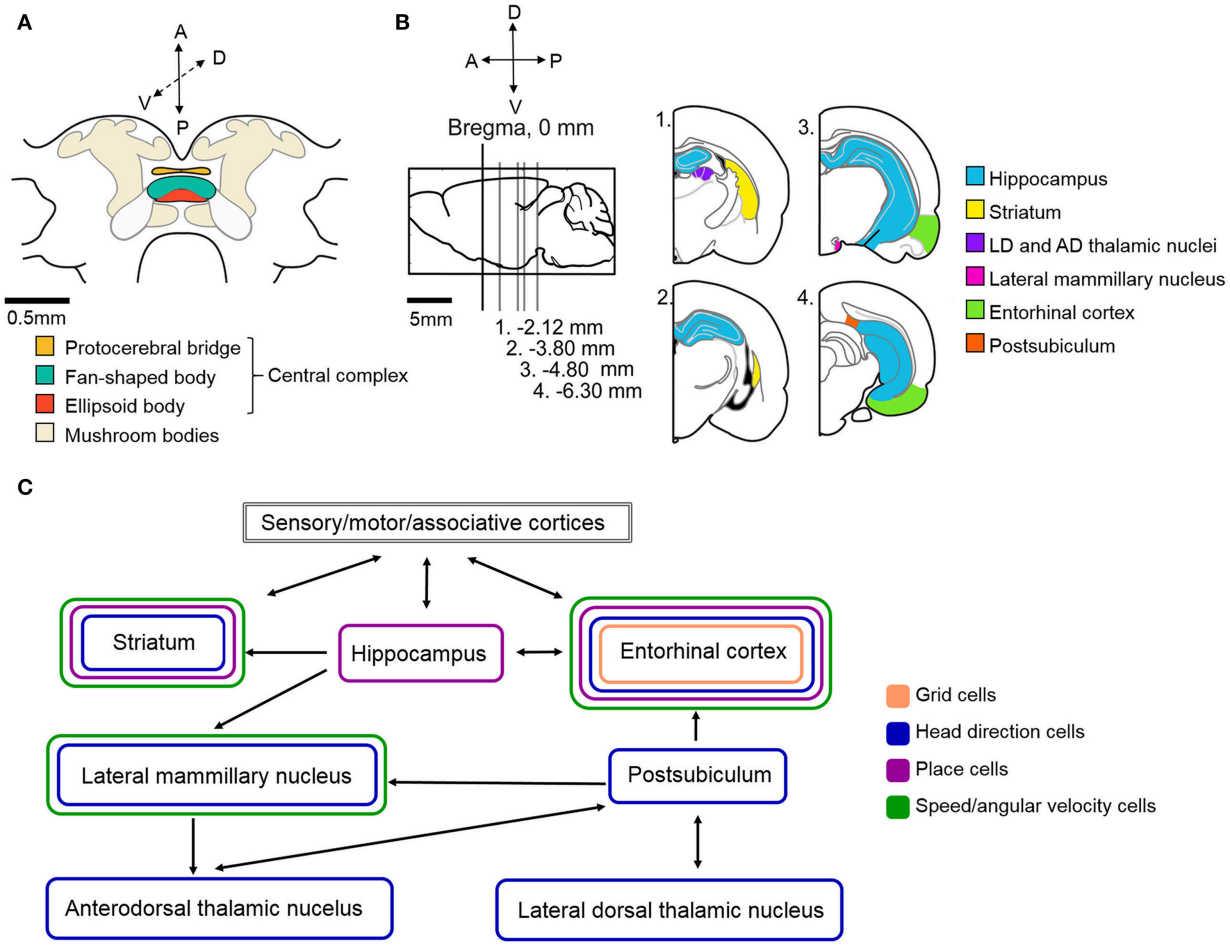
**Schematic illustration of the neural circuitry underlying cockroach and rat navigation and context discrimination. (A)** Schematic illustration of the cockroach central brain with the potential navigation centers color coded (based upon Mizunami et al., 1998). **(B)** Schematic of a rat brain. Sagittal section where Bregma represents 0 mm (marked by black vertical line). Gray lines indicate the location of sections illustrated in the right side of the panel relative to Bregma. Navigation centers are color coded (all rat brain diagrams were created based upon Paxinos and Watson, 1996) **(C)** Arrows indicate the direction of communication between navigation centers in the rat brain. Brain regions are color coded based on the types of spatial cells that can be found in those locations. The exact roles of the above illustrated structures and the connections within the navigation circuit are described in more detail in the text. Based upon (Taube, 2007; Whitlock et al., 2008; Mizumori et al., 2009; Jankowski et al., 2013).

Based on our current understanding of rat navigation, it is thought that the hippocampal formation (including the entorhinal cortex and other parts of the Papez-circuit) and the basal ganglia (specifically the dorsal striatum) in parallel, but differently, process navigational and context-dependent sensory cues to guide behaviors (Figures 4B,C). Within both regions, specialized intertwined networks encode the inner representation of the animal's location, head direction, various aspects of movement (e.g., speed, angular velocity) and, if present, navigational task-related rewards (Mizumori et al., 2009; Penner and Mizumori, 2012).

## Hippocampal formation and related circuits

The hippocampal formation is hypothesized to act as a spatial context discriminator in the rat navigation system. Its role in navigation is to compare the current spatial framework to an expected spatial context. The discrimination process requires access to spatial memory and the ability to detect and encode information about novelty. Novelty in the environment induces exploration or goal-directed navigation, because the current spatial context does not match the expected, or the animal has not yet reached the navigational goal. This process not only induces navigation, but also facilitates learning and memory (Paulsen and Moser, 1998). The hippocampal formation needs to continuously integrate sensory information about the environment, sensory information derived from movement and the current motivational state of the animal as a function of space and time, which translates to the current spatial context. This is achieved by hippocampal *place cells*, that each encode a particular *location* in the environment and also information about behavioral context in one signal (O'Keefe and Dostrovsky, 1971; McNaughton et al., 2006; Yartsev and Ulanovsky, 2013; Figures 4B,C). A hippocampal place cell's preferred location, where the cell's activity increases, is called a place field. Place fields are hypothesized to be the result of the specific spatially and temporally relevant organization of the above listed information. In other words, a place cell's place field contains an abstract description of the animal's current spatial context, which includes information about the environment as well as the inner state of the animal (Mizumori et al., 2009; Penner and Mizumori, 2012). Thus, the comparison between the current and expected spatial context may be achieved with the help of place cells in the hippocampus.

The two most critical pieces of information necessary for navigation are *location* and *orientation*. Orientation in mammals is encoded by *head direction cells* located in various parts of the Papez-circuit, including the postsubiculum, entorhinal cortex, anterodorsal thalamic nucleus, the hippocampal CA1 area and the dorsal striatum (Taube et al., 1990a,b; Taube, 2007; Finkelstein et al., 2011; Rubin et al., 2014; Figures 4B,C). Each head direction cell is tuned to a single preferred head orientation, thus together the network covers the entire 360° environment like a compass. A head direction cell's firing rate reaches the maximum when the animal faces the cell's preferred direction, and as the animal turns away from that angle, the firing rate drops down to near zero almost linearly. Head direction cell firing rate is independent of the animal's location, the head's pitch or roll within ~90° of the horizontal plane, as well as any kind of ongoing behavior, which usually includes walking (Taube, 2007). However, head direction cells in some brain areas also encode *angular velocity*, which results in increased firing rates at the preferred angle when the animal quickly turns its head through this angle, and slightly decreased activity when the turn is slow or the animal is stationary (Taube and Burton, 1995). The sensory cues underlying and forming the head direction signal have been studied extensively in rodents (Taube, 2007). In these studies, rats are placed in a darkened arena with a light cue card placed at some position. Changes in head direction cell preferred angles due to manipulations to the cue card's position suggest that head direction cells establish their preferred orientations primarily based on visual information. Shifting the cue card's position usually leads to corresponding shifts in head direction cell preferred directions, suggesting that the head direction signal may be anchored to visual landmarks (Taube et al., 1990b; Taube and Burton, 1995). The removal of visual landmarks from the environment, or turning off the lights, does not abolish head direction cell firing even when no other allothetic cues (olfactory or tactile) are present, although the preferred angles might drift (Taube et al., 1990b; Goodridge et al., 1998). The directional signal can also be maintained even when the animal is passively rotated around in an arena, indicating that vestibular cues are more critical to the head direction signal than proprioceptive feedback or motor efference copy. The head direction signal is also retained when passive rotations take place in complete darkness, supporting that the head direction system can rely on vestibular inputs when visual landmarks are not available.

Extracellular recordings provide an advantage in these types of studies in that a single electrode can record the activity of multiple cells simultaneously. This technique has been traditionally used in mammalian navigation studies and provided researchers with the opportunity to look at relationships among several head direction cells. The Knierim laboratory provided evidence suggesting that head direction cells, at least within one brain region, might function together as a network (Yoganarasimha et al., 2006). They found that sensory manipulations to the environment, such as landmark removal, result in approximately equal shifts in preferred directions of all recorded cells. However, the amount of the shift is unpredictable, and the neural processes leading to the shift are still not known. Nevertheless, because each cell responds similarly and with equal shifts, we can assume that the specific inputs driving such a change similarly affect all head direction cells in that particular brain region. Thus, head direction cells resemble a coherent neural network where the preferred directions are always a fixed angle apart from each other and perturbations to the environment lead to changes in every individual cell's firing patterns.

The head direction network is a fundamental component in the vertebrate navigation system. Since the two critical pieces of information necessary for navigation are location and orientation, without head direction cells accurate navigation is not possible. Because positional information is independent of orientation, there might not be a direct link between place cells and head direction cells, however to our knowledge, this hypothesis has not been supported or rejected to date.

Another major component of the vertebrate navigation circuits is the grid system (Hafting et al., 2005; Moser et al., 2013; Yartsev and Ulanovsky, 2013; Bush et al., 2015; Rowland et al., 2016). *Grid cells* are principal cells in the medial entorhinal cortex that, similarly to place cells, fire when the animal crosses specific locations within an environment (Figures 4B,C). While place cells only have a single place field where they fire, grid cell firing fields are hexagonally arranged and repeat at regular intervals over the entire environment creating a grid-like structure of place fields. This grid-like firing pattern contains complex spatial information, including *location* in the environment, a regular metric of *distance*, movement related information and likely *orientation*. Because grid cells in different layers of the medial entorhinal cortex span multiple scales and orientations (larger/smaller distances in the grid pattern and different orientations based on external cues), combinations of grid cells can provide information about distance and location in any environment (McNaughton et al., 2006; Rowland et al., 2016). The exact source of positional information and thus the relationship between place cells and grid cells is still unknown, however there is physiological evidence supporting interactions between the two populations of spatial cells (Witter and Amaral, 2004; Langston et al., 2010; Wills et al., 2010; Bonnevie et al., 2013). How grid cells might rely on place cells and *vice versa*, is still not clear (Bush et al., 2014; Dordek et al., 2016).

On the other hand, an elegant study from Winter et al. showed that grid cells rely upon head direction cells to encode orientation (Winter et al., 2015). They lesioned the head direction system located in the anterior thalamic nuclei with a reversible lidocaine injection and found that the inactivation of this orientation signal source disrupts grid cell firing. The animals recovered from the lidocaine injections within ~1.5 h and so did the recorded grid patterns. These data provided the first piece of evidence showing that grid cells receive orientation cues directly from the anterior thalamic head direction network and that the representation of distance, and to some degree, position, is highly dependent on the orientation input from the head direction system.

Another kind of spatial cell located in the entorhinal cortex, *speed cells* (Figures 4B,C), are also thought to provide the rat navigation system with continuous updates during navigation (Kropff et al., 2015). Speed cells encode the running speed of the animal at any given moment during locomotion and their firing rates proportionally increase as the animal increases walking/running speed. Speed cells may provide the grid network with information about speed and distance.

Additionally, *border cells or boundary cells* are hypothesized to encode the shape of the environment that navigation takes place in (Barry et al., 2006; Savelli et al., 2008; Solstad et al., 2008; Lever et al., 2009). They do so by significantly increasing (or decreasing) their firing rate next to specific walls and/or corners of the arena. Border cells are located in several brain areas surrounding the hippocampus, including the entorhinal cortex.

## Spatial code in the basal ganglia

Working in parallel with the above described spatial networks, the dorsal striatum of the basal ganglia is thought to assist the navigational system by evaluating the consequences of behaviors in the current navigational context (Schmitzer-Torbert and Redish, 2004; Penner and Mizumori, 2012). As a result of this analysis, planned motor actions can be fine-tuned to appropriately fit the current context. This process, as well as the motor actions approved by the striatum, have spatial components, which suggests that spatial context processing takes place within the striatum. Information about the environment and the animal's position can be derived from preprocessed sensory information that arrives to the striatum from sensory areas, other associative areas and the limbic system (Mcgeorge and Faull, 1989). Lesion studies showed that impairment of the striatum leads to selective spatial deficits, especially during tasks that require learning (Mizumori et al., 2009). Extracellular recordings in freely behaving animals confirmed that some striatal neurons are sensitive to directional motor components of navigation, such as angular velocity, forward walking speed and navigational context cues, such as a reward's location (Lavoie and Mizumori, 1994; Figure 4). The striatum, similarly to the hippocampus, contains *place cells* that encode the inner representation of the animal's position and other context cues in the environment. In addition to place cells, the striatum also contains *head direction cells*, which encode the orientation of the animal (Mizumori et al., 2000; Figures 4B,C). Because both place cell and head direction cell responses significantly change in rearranged or novel environments, the spatial code in the striatum is thought to be highly context-dependent. This supports the hypothesis that the striatum evaluates behavioral, or in this case, navigational consequences and selects the motor actions that can potentially lead to the desired consequences in a context-dependent manner.

## The neural substrates of navigation in the CX

To what extent can the principles found in rat systems be applied to insects? Clearly, insects do not have brain systems that are anywhere near as large and sophisticated as those found in mammals (Figure 4A shows the schematic of a cockroach brain). Nevertheless, if the mammalian system incorporates critical parts of a navigational system, it is likely that insects, which clearly can perform remarkable navigational feats such as long distance migration by monarch butterflies (Reppert et al., 2010) and foraging by ants (Collett, 2012), have evolved some or all of these solutions. Evidence is accumulating that insects do in fact utilize many of these mechanisms in controlling their movements through complex environments.

A wide range of genetic studies provided evidence for the role of various CX cell types in memory processes with spatial components similar to those observed in the hippocampus. For instance, short-term memory traces for visual pattern elevation and contour orientation were linked to the fruit fly's F5 neurons (dorsal FB neurons) and F1 neurons (ventral FB neurons) respectively (Liu et al., 2006; Pan et al., 2009). Similarly, R2/R4m ring neurons in the EB of flies also serve a role in storing most features of a visual pattern (Pan et al., 2009). Flies with silenced EB ring neurons perform poorly in a detour paradigm (Neuser et al., 2008). In this paradigm, individual flies are placed in the middle of an arena, with similar visual pattern displays on the two opposite sides of the arena, which are removed after the fly crosses the midline. Following the crossing, a distractor target is displayed at a 90° angle compared to the fly's heading. Wild type flies tend to turn toward this new visual target, if it is present for at least 500 ms. When the fly is facing the distractor target, it disappears within 1 s. When wild type flies are left in the arena with no visual targets, they recall their original, pre-distractor heading and start walking in that direction again. Thus, these flies are able to store and recall the position of a former target even though it is no longer present in the environment. Contrary to this, flies with silenced EB ring neurons (R3 and/or R4d) cannot remember their pre-distractor heading, suggesting that these neurons are important components of a spatial working memory circuit (Neuser et al., 2008).

Fruit flies can also perform in a place learning paradigm modeled after the classic Morris water maze, that is most commonly used to study place learning in rodents (Morris, 1981; Morris et al., 1982; Ofstad et al., 2011). The insect version of the maze is a circular arena with heated floor tiles and a single cold tile which serves as a rescue platform, and therefore becomes the animal's goal (Mizunami et al., 1998; Ofstad et al., 2011). When tested in this paradigm, wild type fruit flies quickly learn (one trial, 5 min) to locate the cold tile relative to visual patterns displayed on the arena walls. When the pattern is rotated around, over several trials the flies can learn to associate the cold tile's position with the visual features on the wall. However, individuals with silenced R1 neurons in the EB fail this spatial learning task, even though they can perform normal locomotor and optomotor behaviors, visual pattern discrimination and olfactory learning paradigms. These results indicate that R1 neurons are specifically responsible for some aspect of visually-guided place learning that is independent of basic sensory and locomotor functions (Ofstad et al., 2011).

As described earlier, hippocampal place cells participate in encoding the animal's current and past locations in an environment, thus providing a neural substrate for place learning. Whether any of the above described CX cells have the capacity to integrate environmental and internal context information similarly to place cells, remains to be elucidated. Nevertheless, these genetic studies provide a good starting point for further investigations with different imaging and electrophysiological techniques. In addition to CX circuits, the insect mushroom bodies are also considered an important memory center, which have the potential to contain spatial cells that function similarly to place cells (Mizunami et al., 1998; Heisenberg, 2003). The mushroom bodies do not receive any direct sensory inputs, rather they form a parallel processing loop that receives preprocessed sensory cues, similarly to the hippocampus in the rat brain (Capaldi et al., 1999; Menzel, 2014). It has been suggested many decades ago that the CX and mushroom bodies may play opponent roles in regulating behavior (Huber, 1960). Such parallel processing of sensory information could be the neural substrate of the above described spatial context discrimination (as done by the hippocampus) and evaluation of behavioral consequences in the current spatial context (as done by the dorsal striatum of the basal ganglia). Since in rat systems both the hippocampus and dorsal striatum contain neurons that encode the animal's position, if such cells exist in the insect brain, they could potentially reside in multiple structures as well.

Results from our laboratory indicate that some aspects of movement are also encoded by the CX. Similarly to speed cells in the rat entorhinal cortex, we have reported on single cells in the CX that encoded the speed of locomotion in cockroaches (Bender et al., 2010; Martin et al., 2015). The recorded cells' firing rates strongly correlated with the animal's stepping frequency (Bender et al., 2010). Electrical stimulation through one of the recording electrodes induced walking in stationary animals and increased walking speed in moving animals, although the extent of the areas affected by this stimulation is not clear (Bender et al., 2010; Martin et al., 2015).

Mechanisms similar to head direction coding have been studied extensively in insects that use a CX-based polarized light compass (Heinze and Homberg, 2007; Heinze and Reppert, 2011; Homberg et al., 2011; Bech et al., 2014; el Jundi et al., 2015; Reppert et al., 2016). Yet, the first study providing physiological evidence for general orientation processes was published recently by Seelig and Jayaraman (Seelig and Jayaraman, 2015). The authors used two-photon Ca^2+^-imaging to monitor the dendritic responses of a set of 16 columnar neurons that send projections to 16 columns of the EB in the *Drosophila* CX. Unlike some other insects, the fruit fly's EB is ring shaped, or elliptical, so the columns divide it into 16 radial wedges. EB wedge neurons (also called ring neurons) were targeted, because as described earlier, they have previously been shown to process directional visual information and play a role in feature detection (Neuser et al., 2008; Pan et al., 2009; Seelig and Jayaraman, 2013). During the experiments, fruit flies were head-fixed, but they were free to walk on an air-suspended Styrofoam ball in an LED arena. The arena was part of a closed-loop system, where the fly's movements on the ball controlled the position of the projected image on the LED panels. At certain headings relative to the displayed pattern's position, active cells formed a so-called “activity bump,” wherein projections going to approximately 5–6 wedges would show increased activity. Whenever the fly changed its heading, the activity bump in the EB rotated as well. Importantly, any kind of visual scenery evoked this specific response, ranging from a single vertical stripe to more complex visual features. This indicates that the neurons were not encoding the visual information itself, but rather the animal's orientation relative to the visual landmark(s). By varying the closed-loop gain that matched the ball's rotational movements to the visual landmark's movements, they observed that CX activity integrated visual cues more heavily, than self-motion cues. Experiments conducted in darkness revealed similar results. The flies were able to maintain the EB wedge neuron activity bumps with no visual cues, but only for a limited period of time. This indicates, that the fly navigation system accumulates error over time when the only updates on the fly's relative orientation come from walking, thus proprioceptive feedback and motor efference copy. This was the first study that provided evidence for the CX's role as a navigation center with a compass-like function that integrates sensory information about the animal's orientation and through unknown downstream targets, guides movements accordingly.

We further extended the results from the fly experiments by adopting some of the classical methods used in rat head direction cell studies and applying them in our experiments on cockroach CX function (Varga and Ritzmann, 2016). We used extracellular recordings to gain insight into how single neurons in the CX might contribute to the head direction signal and to draw more direct comparisons between the neural strategies underlying rodent and insect head direction coding.

Restrained cockroaches with a fixed head-body axis were implanted with a tetrode wire bundle and placed on a computer-controlled platform in the middle of a dark uniform arena with a single, conspicuous visual cue on the wall. We rotated the animals around in 30° increments and analyzed the changes in CX neuron activity displayed during the 10 s immobile periods between the rotational steps. We found that single neurons in both the FB and EB encoded head direction and among all neurons, the entire 360° environment was represented equally. Some of the recorded neurons encoded head direction with similarly narrow tuning as observed in rat head direction cells, while the majority of them were broadly tuned to their preferred angles. These tuning patterns were reminiscent of the tuning characteristics observed in polarized light studies (Heinze et al., 2009). However, unlike those studies, here the landmark cards were blocked from the insects view for some of the angles, suggesting that orientation was being coded rather than a direct response to visual cues.

**Figure 5:**
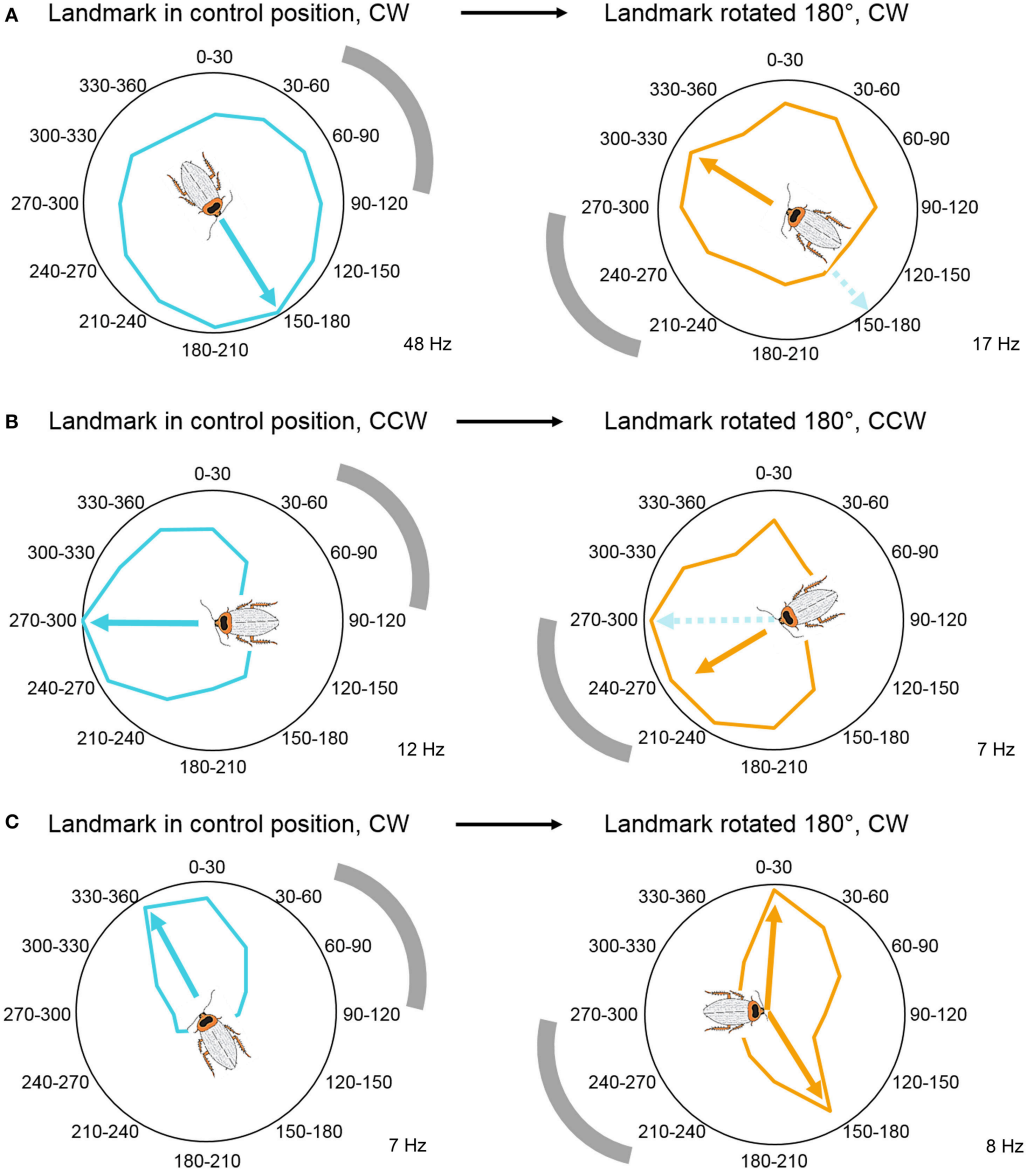
**Visual landmark position determines head direction coding. (A)** Head direction coding tuned to allothetic cues, wherein angle-modulated units follow the shift in landmark position by shifting their peaks. Example unit's mean firing rate over 6 trials, illustrated in a circular plot. **(B)** Head direction coding tuned to idiothetic cues, wherein angle-modulated units persist to encode the original preferred angle. Example unit's mean firing rate over 4 trials, illustrated in a circular plot. **(C)** Bimodal responses during landmark rotation trials. These units developed a second peak in response to the new landmark position, while the original peak persisted to encode the peak from the control trials. Example unit's mean firing rate over 6 trials, illustrated in a circular plot. Gray arch indicates the position of the visual landmark on the wall of the cylindrical arena. All examples were modulated by head direction, *p* < 0.05 Rayleigh test. The cockroach's preferred direction is marked by arrows and the cartoon cockroach's heading (cyan, preferred direction in control trials; orange, preferred direction in landmark-shifted trials). Maximum average firing rates (Hz) of the example units are marked in the right bottom corner of each panel. Modified with permission from Varga and Ritzmann (2016).

Through manipulating the visual cue's position in the arena, we established that, similarly to rat head direction cells, some CX neurons are anchored to the visual landmark's position and encode the animal's relative orientation compared to this cue (Figure 5A). This result is in accordance with Seelig and Jayaraman's findings where the EB activity bump rotated with the visual landmark (Seelig and Jayaraman, 2015). However, we also recorded from neurons that did not shift their peak firing rates when we shifted the position of the visual landmark (Figure 5B), and neurons that encoded two preferred angles after the landmark shift (Figure 5C). These results indicate that some cells in the cockroach's CX compass may rely upon movement-derived idiothetic cues (a process called path integration), even when a visual landmark is available to the animal. Visual landmark removal and experiments with head-covered landmark naïve animals revealed similar results. Because of our passive rotation experimental design, we know that these neurons encoded head direction without any access to proprioceptive feedback or motor efference copy. This finding suggests, that insects, similarly to mammals, have access to vestibular-like sensory inputs, which might directly impact neurons in the CX. Such angular velocity signals could potentially originate from the Johnston's organs at the base of the antennae, but physiological evidence supporting this hypothesis remains to be uncovered (Kamikouchi et al., 2009; Yorozu et al., 2009; Matsuo et al., 2014). Interestingly, when compared to fruit flies, cockroaches did not accumulate a lot of error during landmark-deprived trials. This difference between the results of the two studies could stem from the different ecology of the two model animals. Cockroaches are nocturnal animals that have limited access to visual landmarks and might need to rely on idiothetic cues more often than diurnal flies. However, another explanation is that, although the fruit flies in the closed-loop experiments received proprioceptive feedback and motor efference copy from the legs, their heads were fixed therefore they did not have access to vestibular-like inputs unlike the cockroaches in our rotation experiments. Thus, it is possible that, similarly to mammalian navigation systems, insects primarily rely upon vestibular-like cues rather than leg-derived movement information in the dark and other landmark-deprived situations.

Although these studies provided detailed evidence for orientation coding (not based on specialized sensory cues) in insects, the question whether these cells are “real” head direction cells remains to be addressed. One important characteristic of mammalian head direction cells is that they have the capacity to encode orientation in any environment, completely independent of the animal's *location* in that environment (Taube, 2007). Thus, the above described compass cells will need to be tested in a range of environments in freely behaving animals to determine the effect of *location*, as well as novelty on the head direction signal.

**Figure 6:**
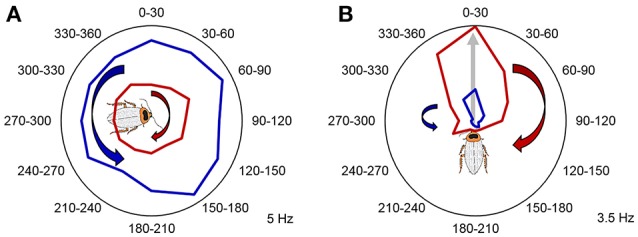
**Past rotation direction affects CX unit firing rate during the stationary epochs. (A)** Example unit not modulated by angle increased its firing rate following clockwise rotations (*p* < 0.05, two-tailed paired *t*-test). Mean firing rate during counterclockwise rotations is marked with red, while mean firing rate during counterclockwise rotations is marked with blue. **(B)** A representative example of a CX unit that significantly encoded a preferred head direction and increased its relative firing rate during the stationary epochs following clockwise rotations. *P* < 0.05 for both Rayleigh test and two-tailed paired *t*-test. Mean firing rate during clockwise rotations is marked with red, while mean firing rate during counterclockwise rotations is marked with blue. The preferred angle of this unit is indicated by the gray arrow and the cartoon cockroach's heading. Maximum average firing rates (Hz) of the example units are marked in the right bottom corner of each panel. Modified with permission from Varga and Ritzmann (2016).

In addition to the animal's position and head orientation, adaptive navigation also depends on spatial contextual cues, such as a navigational goal, a certain component of a navigational task, or relative movement direction (clockwise vs. counterclockwise; left vs. right). As mentioned earlier, these navigational context cues can be encoded by the dorsal striatum in mammals. The navigation circuits in the striatum then may use these cues to direct and shape motor commands (Mizumori et al., 2009). Additionally, the hippocampal-entorhinal navigation circuits can also encode and utilize such contextual information and use it in spatial memory and context discrimination processes (Penner and Mizumori, 2012). To test whether the CX plays a role in storing spatial contextual information, we rotated the animals in both clockwise and counterclockwise directions in a counterbalanced manner. We found that in addition to the compass cells, both the FB and EB contain neurons that encode the rotation direction history of the animal, by increasing or decreasing their firing rates after the rotations (Figure 6). Movement direction is a spatial context cue that is independent of the specific head orientation of the animal. Similarly to mammalian systems, spatial contextual information and head direction may be utilized in spatial memory and in shaping motor commands in downstream circuits.

## Motor control from the CX

In order for the information described in the previous sections to guide foraging movements, the CX must be able to produce or influence motor commands. To examine this aspect of behavioral control, we performed a series of experiments that involved multi-unit extracellular recordings in cockroaches that were either tethered or moving free in an arena. These experiments clearly demonstrated motor control properties recorded in the CX.

Our initial recordings were performed in cockroaches tethered over a lightly oiled plate (Bender et al., 2010). In these experiments single tetrodes were constructed out of bundles of fine insulated wires (Guo et al., 2014). The wires were either plated with copper or dipped in a fluorescent dye so that their recording location (but not the individual cells) could be identified histologically after the experiment. Cockroaches walk normally in the oiled plate tether and will spontaneously change walking speed. Plots of walking speed and rate of action potential activity in many CX neurons were strongly correlated. Moreover, delaying the functions that described neural activity increased the correlation with walking speed, suggesting that CX activity changes typically preceded altered walking speed. Also, stimulation through the same electrodes evoked similar increases in step frequency.

**Figure 7:**
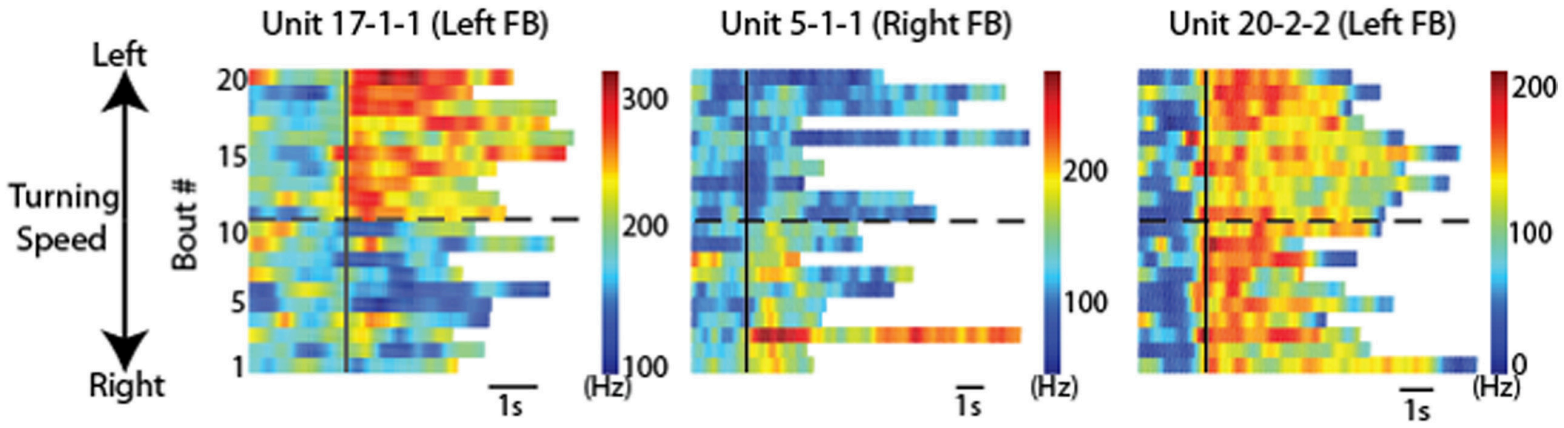
**CX units responded to locomotion in a directionally biased manner**. Raster plots of 20 bouts of locomotion for three units. Each row is one bout and the color indicates the firing rate. For each graph, the solid black line indicates the start of each bout. Bouts of left turning are above the dashed black line and bouts of right turning are below it. For bouts of left turning, the higher the bout number, the higher the average turning speed. For bouts of right turning, the lower the bout number, the higher the average turning speed. Note the changes of firing rate after locomotion start as a function of locomotion direction. Individual units are named according to preparation, tetrode and unit numbers (e.g., “unit 1-2-3” indicates preparation 1, tetrode 2, unit 3). Reproduced with permission from Guo and Ritzmann (2013).

Directional turning was examined by placing a wired cockroach on an air-suspended Styrofoam ball (Guo and Ritzmann, 2013). A rod was placed near the cockroach's head. As had been demonstrated earlier, cockroaches will often explore a similar rod with their antennae and turn to examine it further (Okada and Toh, 2000). Before the cockroach turned to the left or right, activity changes were noted in FB recordings (Figure 7). This pattern of activity change had a distinctively biased directionality. Recordings made in the left FB found cells that increased activity prior to only left turns, but never found cells that only signaled right turns and vice versa. In addition to these biased responses, cells were also found on both sides of the FB whose activity preceded movement in either direction.

**Figure 8:**
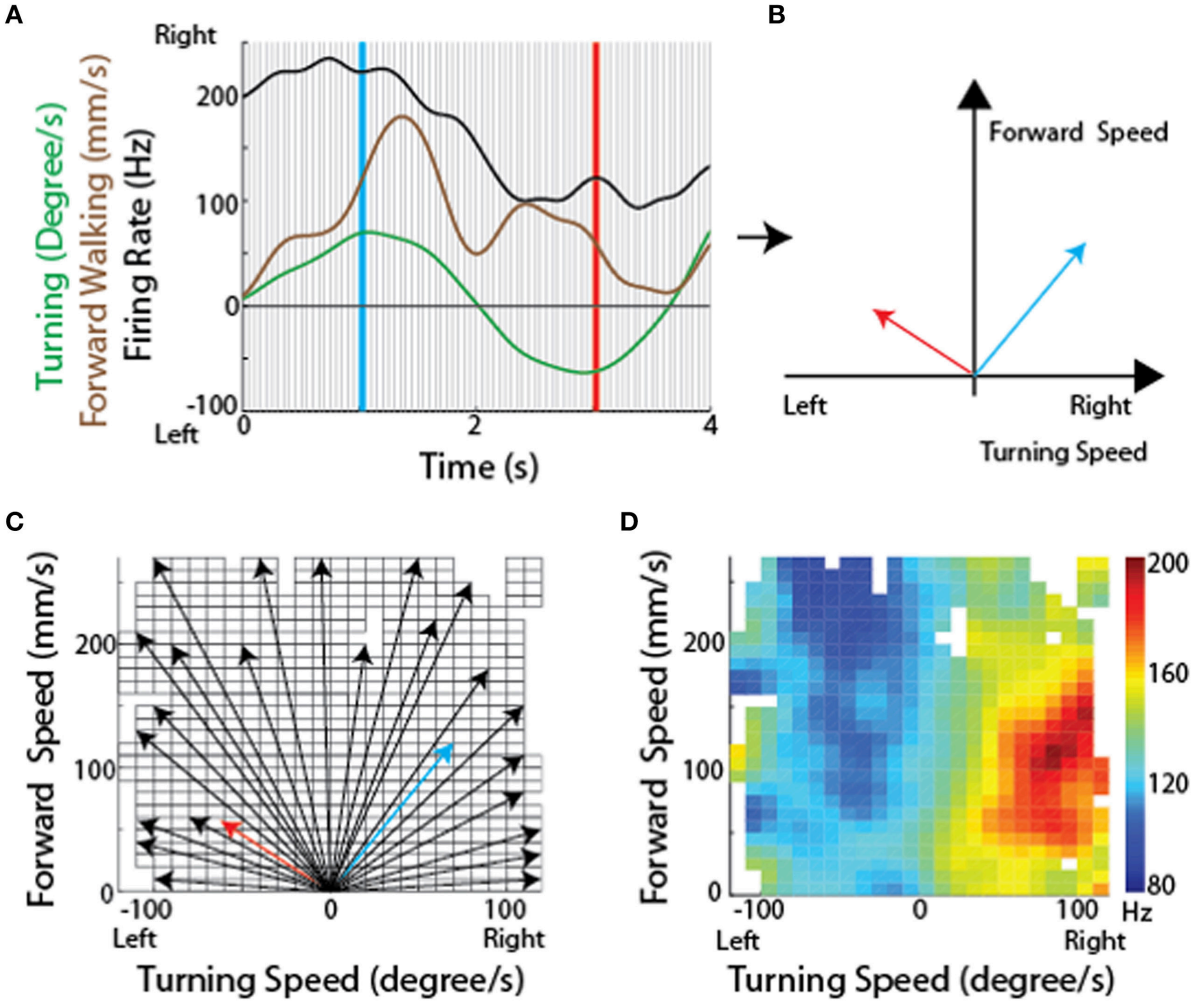
**Methodological concept for generating firing rate maps in tethered insects. (A)** For every recording session, forward and turning speed as well as spike times of each unit were smoothed using a Gaussian kernel with a standard deviation of 150 ms. Each recording session was divided into non-overlapping 50 ms long sections (between individual gray lines). **(B)** For each divided section, a velocity vector was generated by averaging forward and turning speed within that period. Firing rate for each velocity vector was also calculated. The blue and red vectors were obtained from the blue and red lines, respectively, in **(A)**. **(C)** All velocity vectors were binned into a forward walking speed vs. turning speed graph (10 mm/sec for forward walking speed and 10 deg/sec for turning speed). Only some of the vectors, including the two vectors in B, are shown here. **(D)** A firing rate map was generated by overlaying the averaged firing rate for each bin, obtained by averaging all the firing rates whose corresponding velocity vectors fell into that bin. Reproduced with permission from Guo and Ritzmann (2013).

Because the optical sensors that monitored ball movement indicated changes in forward (translational) movement as well as right-left rotational movement, these data could describe two dimensional maps of the movements with which each cell's activity was associated. To generate these maps, we plotted the firing rate for each recorded CX neuron along with forward walking speed and turning. The data were then divided into bins and for each bin the translational and rotational value described a vector (Figures 8A,B). At the tip of each vector, we placed a square that was color coded according to the firing rate of that neuron. When this was completed for the vectors describing each bin, the data defined a two dimensional map of the types of movements with which each cell's activity was associated (Figures 8C,D).

**Figure 9:**
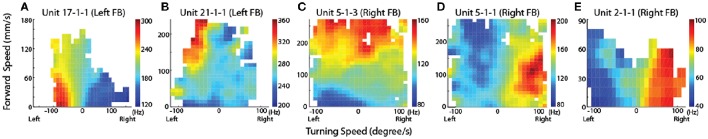
**CX units are tuned to self-motion**. Firing rate maps for locomotion initiated by antennal contact with the rod for representative CX units. The x-axis is the turning speed and the y-axis is the forward walking speed. Positive turning speed indicates right turning and negative turning speed indicates left turning. CX units showed discrete locomotion-related firing fields, such as left turning irrespective of forward walking speed (**A**, *Z* = 3.59, *P* < 0.01), forward walking to the left (**B**, *Z* = 4.08, *P* < 0.01), forward walking irrespective of turning speed (**C**, *Z* = 5.73, *P* < 0.01), forward walking to the right (**D**, *Z* = 6.00, *P* < 0.01), and right turning irrespective of forward walking speed (**E**, *Z* = 2.33, *P* < 0.01). Reproduced with permission from Guo and Ritzmann (2013).

The resulting maps identified cells in the left FB that were associated with slow left turns (Figure 9A) while others were associated with only fast left turns (Figure 9B). Neurons were also found associated with fast turns to either direction (Figure 9C), but right turn biased cells were only recorded in the right FB and left turn biased cells in the left FB (Figures 9D,E). As with the oiled plate experiments, stimulation through the recording electrodes generated turns that were consistent with the recording biases. That is stimulation in the left FB consistently generated left turns while stimulation in the right FB generated right turns.

**Figure 10:**
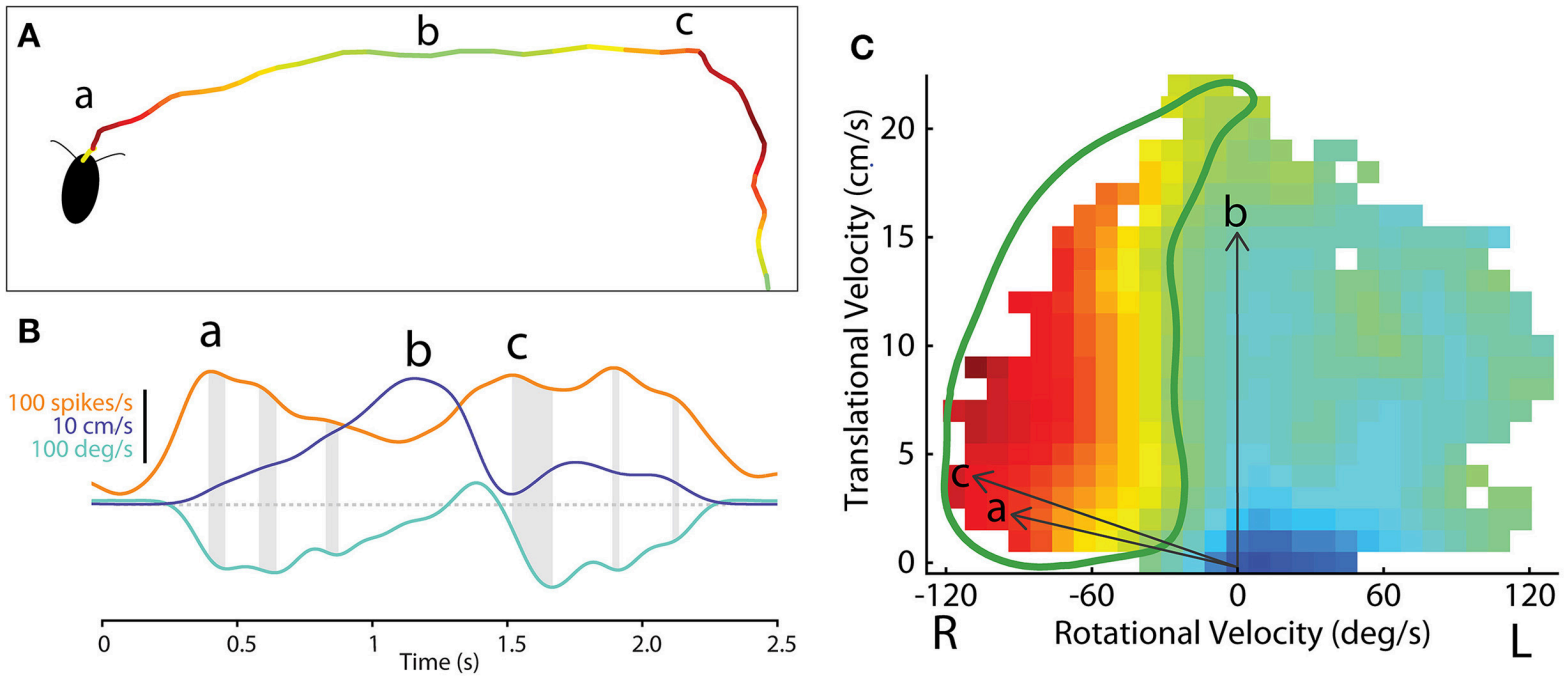
**Methodological concept for generating firing rate maps based on locomotor-related activity in the CX of freely-moving cockroach. (A)** The path of a cockroach exploring the open arena. Color indicates firing rate of an example central-complex neuron during each segment of movement. **(B)** Smoothed firing rate of a central-complex neuron (orange), translational velocity (purple) and rotational velocity (cyan) of the animal during the bouts indicated at (a) and (b) and (c) in section A. Gray shading indicates the delay between peaks in the firing rate and peak rotational velocity of the resulting movement bout. **(C)** The firing rate for a single neuron is related to direction of movement in a manner similar to that used for tethered experiments (Figure 8). Here vectors of translational velocity vs. rotational velocity were created from video data (e.g., vectors a, b and c from time points indicated in **B**). At the tip of each vector a color coded box indicates the firing rate at that point in time. When this is done throughout a bout, a raw map of activity relative to motion is constructed. The firing rate map is then smoothed and gap-filled in 2 dimensions. Contour lines are then constructed from 0 to 100% of the maximum firing rate of this cell (not shown here). The 50% contour (thick, green line) is taken to represent the characteristic activity of the cell relative to movement direction. Adapted with permission from Martin et al. (2015).

The observations described above, taken with tethered insects, are very useful, but they represent open loop movements rather than natural foraging behaviors. To get closer to normal movements, we adapted our recording techniques to allow them to be performed in freely moving cockroaches (Figure 10A; Guo et al., 2014; Martin et al., 2015). Here the cockroach's actions were recorded with video cameras and moment-to-moment movements were again separated into forward (translational) and left-right (rotational) movements throughout the insect's track. As with the tethered experiments, we could then relate spike frequency of CX neurons to those actions (Figure 10B).

**Figure 11:**
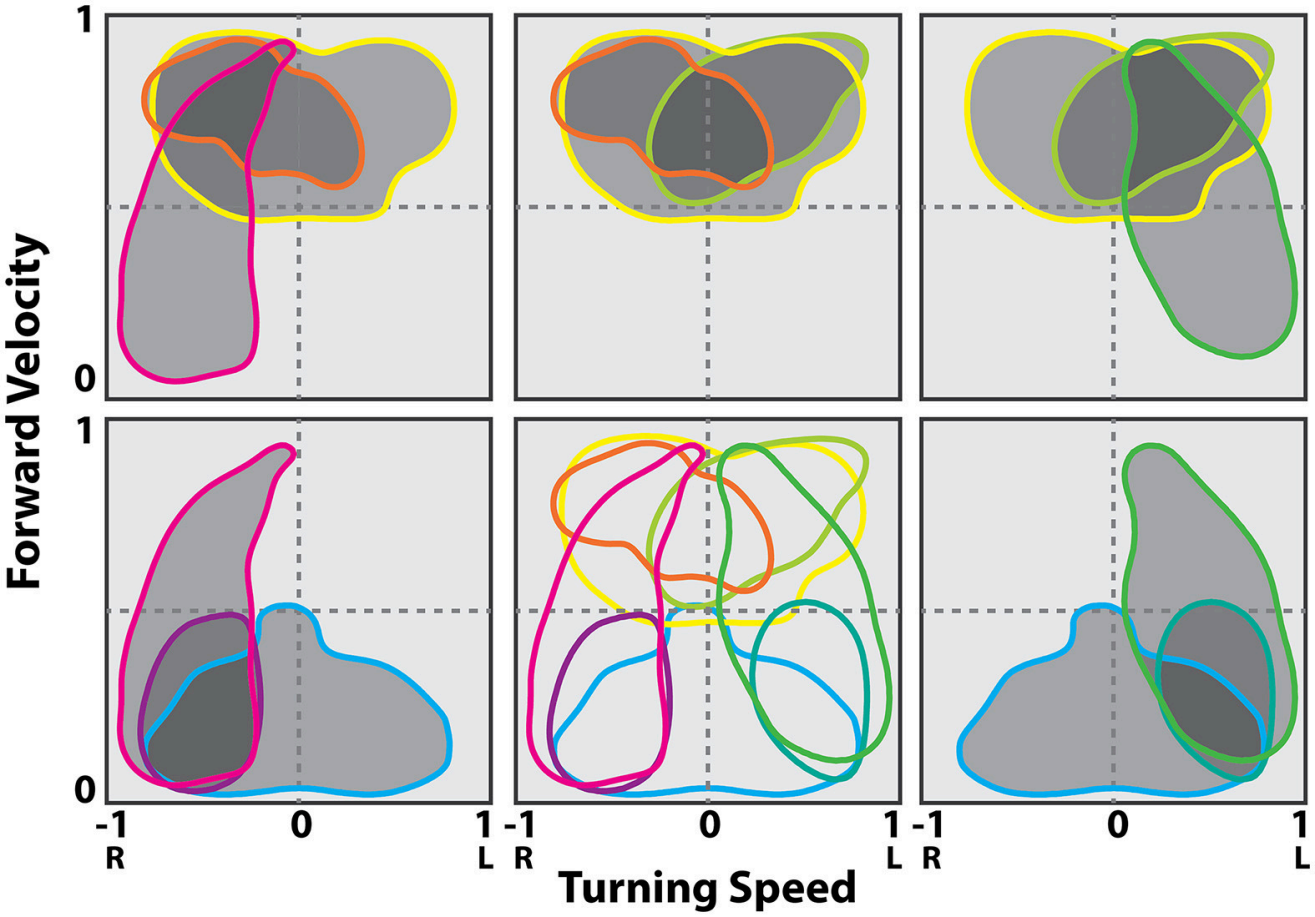
**Contour lines in the translational and rotational velocity axes at the 50% of maximum firing rate levels from the firing rate map at the best delay (location of the peak in the stimulus kernel)**. Cells shown are a representative sampling of cells spanning the range of observed selectivity. Shading indicates groups of cells in a possible population selected for fast turns to the right (gray), fast turns to the left (yellow) and slow turns to the right (cyan). Modified with permission from Martin et al. (2015).

These data could then be plotted as two dimensional motor maps as we did in the tethered experiments. We then smoothed and gap filled these maps and plotted contours that encapsulated 0–100% of maximum firing rate. The 50% contour was taken as characteristic for that cell and could then be compared to other neurons in the same and other insects (Figure 10C). A plot of all 50% firing contours describes a population code for movement in two dimensions (Figure 11). These contours encapsulate the entire set of movements that the cockroach could make in two dimensions. As with the tethered experiments the majority of changes in firing frequency preceded changes in movement. A few effects did follow changes in movement and some cases were recorded where activity changed both before and after a movement was executed.

As with the navigational system study, these data point to yet another similarity between insect and mammalian systems. Population codes for movement have been described in many mammalian motor control systems. Perhaps one of the earliest examples of population codes was demonstrated for arm movements in monkeys (Georgopoulos et al., 1988; Schwartz et al., 1988; Georgopoulos, 1995). In those studies, monkeys were trained to move their arms in three dimensional space from a starting position to a target location while neurons were recorded in the motor cortex. Many of these neurons were found to have a directional bias in that they fired at maximal levels during arm movements in a particular direction with fall-off in other directions. As with the cockroach data, the entire population of preferred directions covered the entire movement space. For any arm movement, a vector sum of the firing rate of 475 cells showing that direction could accurately predict the actual arm movement.

How do CX neurons come to affect turning movements? Much of the output from the CX projects to the lateral accessory lobes where they encounter neurons that descend to the thoracic ganglia (Heinze and Homberg, 2008). When the cockroach turns, the motor activity associated with leg movements must change. In particular the middle leg on the inside of the turn switches from extending during stance to extending during swing. That leg then touches down and pulls the body through the turn (Mu and Ritzmann, 2005). In stick insects, walking movements of individual leg joints are coordinated by inter-joint reflexes (Akay et al., 2001, 2004). As the insect turns or walks backward, many of these reflexes reverse (Akay et al., 2007; Hellekes et al., 2012). Similar inter-joint reflexes have been found in cockroach middle legs and reversal of these reflexes occurs when descending activity is removed through bilateral ablation of cervical connectives (Mu and Ritzmann, 2008). These observations suggest that CX circuits could alter leg movements by affecting descending activity which, in turn, orchestrates specific reflex reversals.

**Figure 12:**
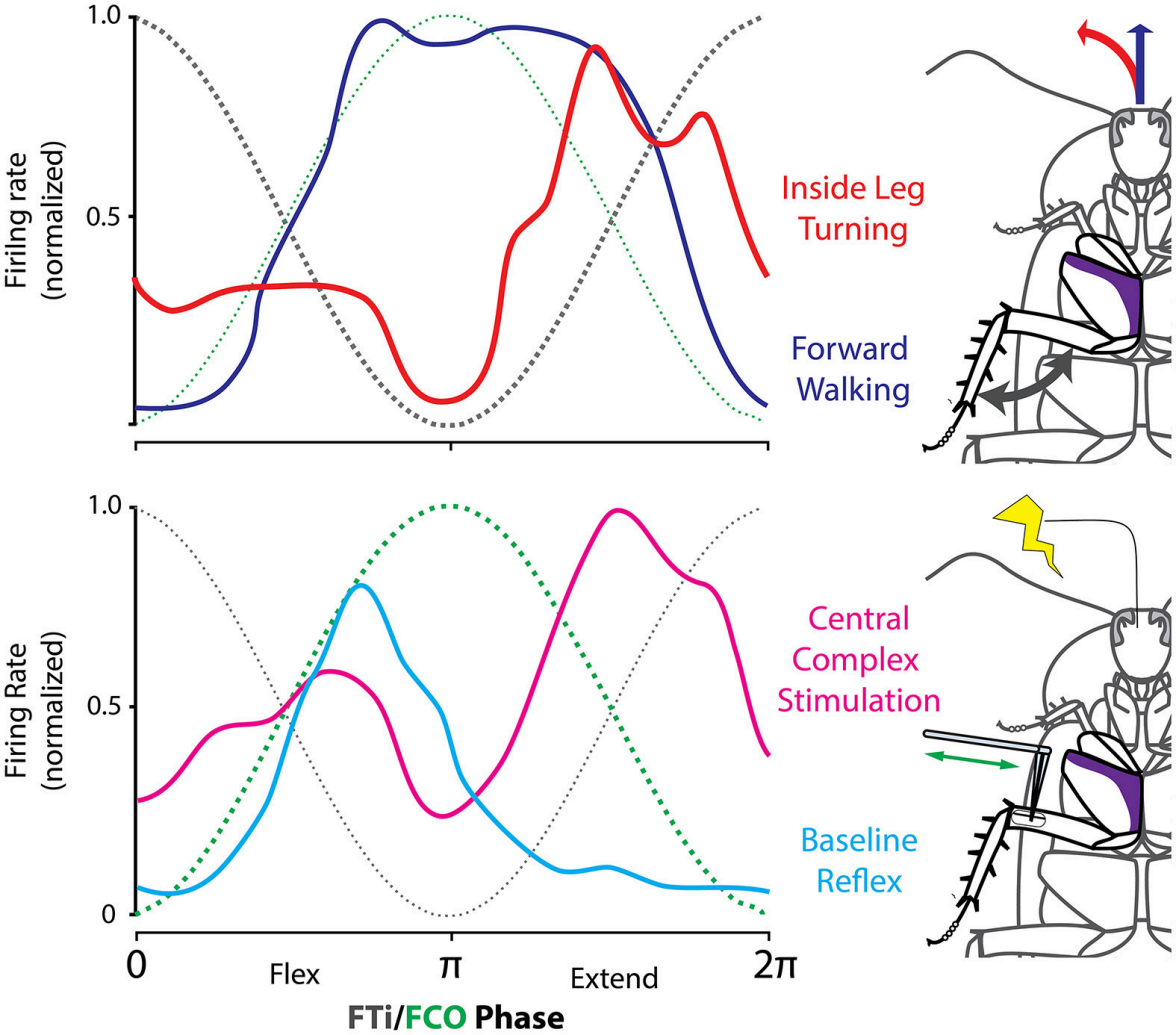
**CX stimulation evokes reflex reversal. (Top)** The firing rate distribution of Ds neurons as a linear histogram, with the FCo (green) and FTi (black) phase indicated taken during spontaneous forward walking (blue) and turning (red) evoked by stimulation through CX electrodes in a freely moving cockroach. **(Bottom)** Same insect was moved to a preparation dish and the FCo was exposed by dissection. Here Ds activity is shown in response to FCo extension and relaxation with (red) and without (blue) stimulation through the same electrodes in the CX that evoked turning in the top record. Modified with permission from Martin et al. (2015).

To test this hypothesis, we identified a subset of subjects in our arena experiments in which stimulation through the CX tetrodes consistently evoked turning movements in a particular direction. This meant that we could identify the leg that consistently represented the inside leg of turns evoked by CX stimulation in these subjects. In these experiments, we also recorded EMGs from middle leg coxal muscles. As in previous tethered experiments, the slow depressor of the coxa (Ds) changed its firing pattern dramatically when the cockroach transitioned between the patterns associated with forward stepping and to that of inside leg turning (Figure 12).

With this in mind, we moved the cockroaches from the arena to a preparation dish with the CX electrodes in place. We then exposed the femoral chordotonal organ (FCo) that monitors femur-tibia joint movements (Figure 12). Without CX stimulation, FCo stretch and release generates Ds activity that is consistent with the initial activation seen in forward walking. When FCo stretch and release was repeated in conjunction with CX stimulation, the Ds reflex reversed to follow a pattern very similar to that seen during turning.

Of course, movement in the horizontal plane represents only a portion of the cockroach's, or other insects', foraging behaviors. Cockroaches readily climb over substantial blocks, walk up walls and can even walk inverted along ceilings. Does that pattern of CX motor control change under altered context? To test this we moved cockroaches with the tetrodes in place from the arena to a runway that included a large block. This forced the cockroach to execute climbing movements in order to proceed. We plotted the relationship between step frequency and rate of action potentials for individual CX neurons taken during horizontal walking and climbing. Some of these cells showed no change in this relationship, but others changed dramatically. Some retained their slope but shifted upward. Others altered slope and some were even found to reverse the function so that they decreased activity as step frequency increased. This observation suggests that the population code seen in Figure 11 for horizontal walking is dynamic in that it can be greatly modified when the cockroach starts to climb. Other behaviors would be expected to generate further alterations in this population code.

## Conclusions

The experiments described here strongly suggest that the CX plays a pivotal role in controlling insect behavior. The specifics of this role will vary from insect to insect and behavior to behavior. As a result, the effects seen in various insects will vary with the behavioral niches each species inhabits. Thus, migratory insects like monarch butterflies and locusts will tap into polarized light maps to control flight movements as they make long distance flights. Foraging insects, like stick insects and cockroaches will utilize visual and tactile cues to move through their environments toward targets and away from threats. Nocturnal vs. diurnal insects will use appropriate cues to guide their movements (el Jundi et al., 2015). Predatory insects would be expected to use these tools to target prey and guide stalking movements.

Our discussion also demonstrates that many of the properties associated with navigation and motor control in mammals can be found in insect CX data. Whether this is a matter of convergence or, as has been suggested by others, deep homology (Strausfeld and Hirth, 2013), the ramifications are important. At the very. least, it suggests that there are neural properties that are essential for effective solutions of navigation and motor control.

## Author contributions

AV performed head direction experiments and described head direction work. NK performed visual CX experiments and described CX visual work. JM performed CX motion experiments and described this work. PG performed tethered motion experiments and described this work. RR Oversaw all work as Principle Investigator of Laboratory and oversaw manuscript.

### Conflict of interest statement

The authors declare that the research was conducted in the absence of any commercial or financial relationships that could be construed as a potential conflict of interest.
